# Supplementary material for: Metabolic adaptations to acute glucose uptake inhibition converge upon mitochondrial respiration for leukemia cell survival
Source: Cell Commun Signal. 2025 Jan 25;23:47. doi: 10.1186/s12964-025-02044-y (PMC11762851; doi:10.1186/s12964-025-02044-y)
Supplement: Supplementary file 1 — Supplementary Material 1. [file 12964_2025_2044_MOESM1_ESM.pdf]

Supplemental Figure 1

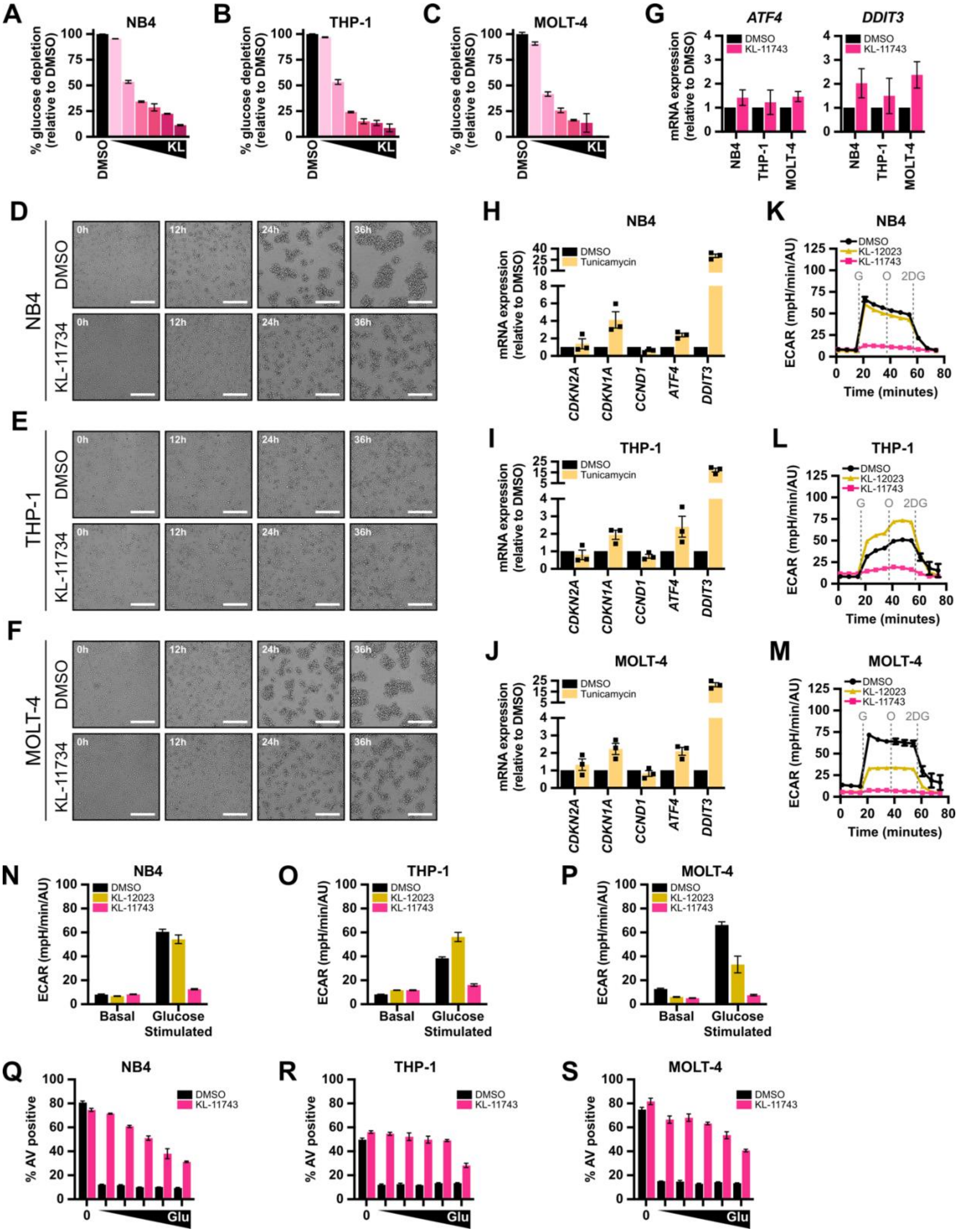

Supplemental Figure 1. KL-11743, but not KL-12023, inhibits glucose uptake and glycolysis in hematological cells.

(A–C) NB4, THP-1, and MOLT4 were treated with DMSO or KL-11743 (10, 100, 250, 500, 1000, 1500 nM) for 24 h. Different ranges were tested for each serum lot. Glucose concentrations from the cultured media were determined and presented as % glucose uptake relative to DMSO. Error bars are the SEM.

(D–F) Representative images of NB4, THP-1, and MOLT-4 cells treated with KL-11743 (500 nM) for 0, 12, 24, and 36 h. Scale bar: 300 μm.

(G) NB4, THP-1, and MOLT-4 were treated with DMSO or KL-11743 (500 nM) for 8 h, and total RNA was harvested. The fold change of transcripts for *ATF4* and *DDIT3* (CHOP) was determined by real-time qPCR. Expression was normalized against *18S*.

(H–J) NB4, THP-1, and MOLT-4 were treated with DMSO or tunicamycin (100 ng/ml) for 8 h, and the indicated genes were determined by real-time qPCR. Expression was normalized against *18S*. Data are presented as the mean of 3 replicated experiments ± SEM.

(K–M) NB4, THP-1, and MOLT4 were treated with DMSO, KL-11743 (500 nM), or KL-12023 (500 nM) for 24 h and ECAR was measured following an Agilent XF Glycolysis Stress Test. G: glucose (10 mM), O: oligomycin (1 μM), 2DG: 2-deoxy-D-glucose (50 mM). Error bars are the SEM.

(N–P) Data from *K–M* presented as basal and glucose-stimulated ECAR. Error bars are the SEM.

(Q–S) NB4, THP-1, and MOLT4 were cultured in serum-free media for 24 h, then supplemented with glucose (0, 0.1, 0.5, 1, 5, 10 mM) ± KL-11743 (500 nM) for 24 h. Apoptosis was measured by AV labeling and flow cytometry. Error bars are the SEM.

Supplemental Figure 2

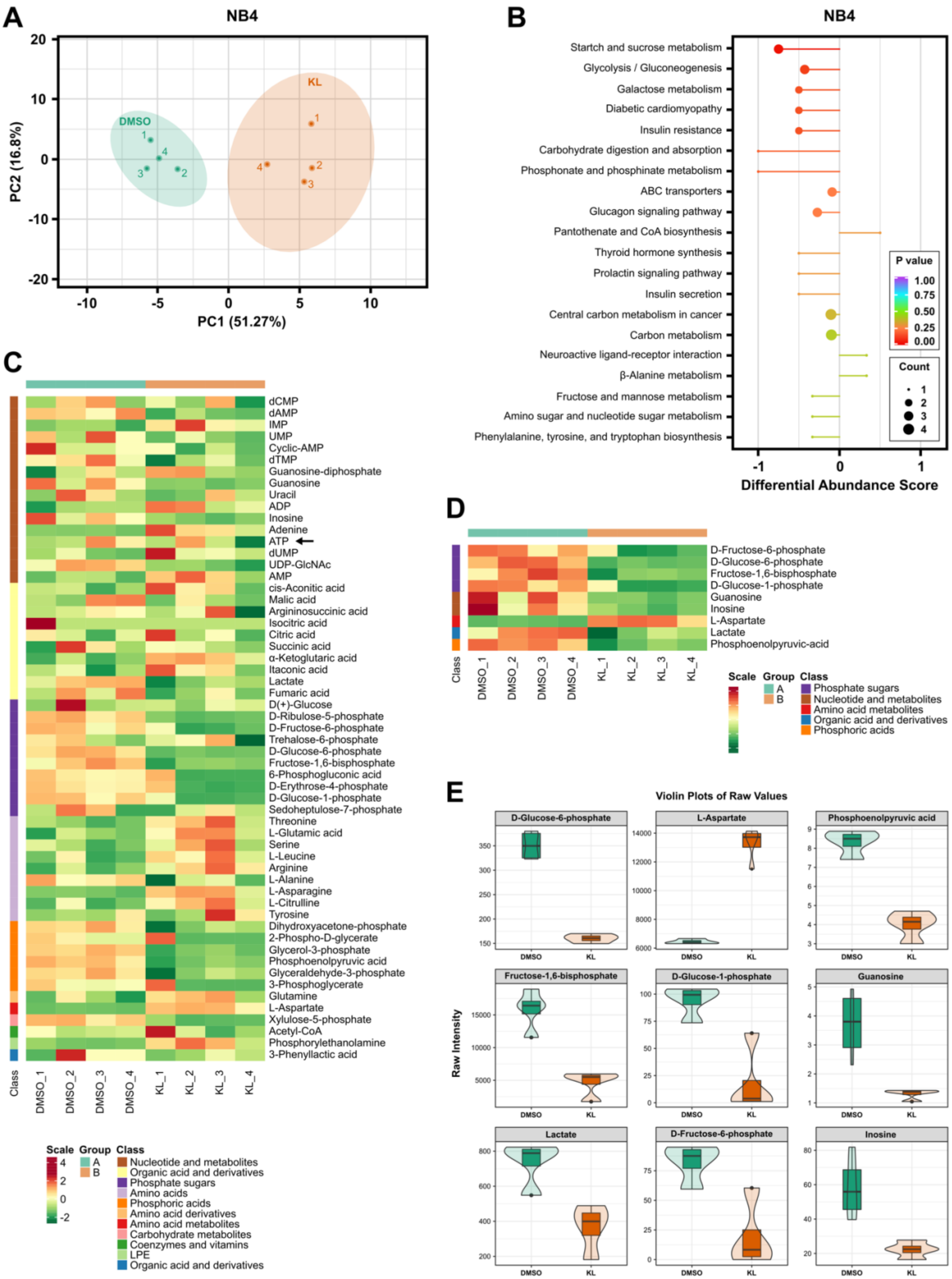

Supplemental Figure 2. Targeted metabolomics of NB4 treated with KL-11743.

(A) NB4 cells were treated with DMSO or KL-11743 (500 nM) for 24 h. Samples were subjected to LC-MS/MS and PCA detecting 57/68 energy related metabolites within the MetWareBio Energy Metabolism module. Unsupervised PCA was performed using prcomp within R; data were unit variance scaled before unsupervised PCA.

(B) KEGG pathway enrichment analysis was conducted based on the annotation results. The size of the dots in the figure represents the number of significantly different metabolites enriched in the corresponding pathway. The X-axis represents the Rich Factor and the Y-axis represents the pathway. The color of points reflects the p-value. The darker the red, the more significant the enrichment. The size of the dot represents the number of enriched differential metabolites.

(C) Hierarchical Cluster Analysis was used to cluster the samples. X-axis indicates the sample name and the Y-axis are the metabolites. Group indicates sample groups. Z-Score indicates the relative quantification of each metabolite with red representing higher content and green representing lower content. Cluster analysis was performed on both metabolites (vertical cluster tree) and samples (horizontal cluster tree). Heatmap was drawn by R software Pheatmap package.

(D) Heatmap of different metabolites. The X-axis shows the name of the samples, and the Y-axis shows the differential metabolites. Different colors in the heatmap represent the values obtained after normalization and reflect the level of relative quantification. The darker the red, the higher the quantification. In contrast, the darker the green, the lower the quantification. The colored bar on top depicts sample groups.

(E) Violin plots display data distribution and probability density. X-axis refers to sample, and the Y-axis refers to content. The box in the middle represents the interquartile range, and the middle box represents the 95% confidence interval. The black horizontal line is the median, and the outer shape represents the distribution density of the data. The figure shows the result of the top 9 differentially expressed metabolites with the largest Log_2FC value.

Supplemental Figure 3

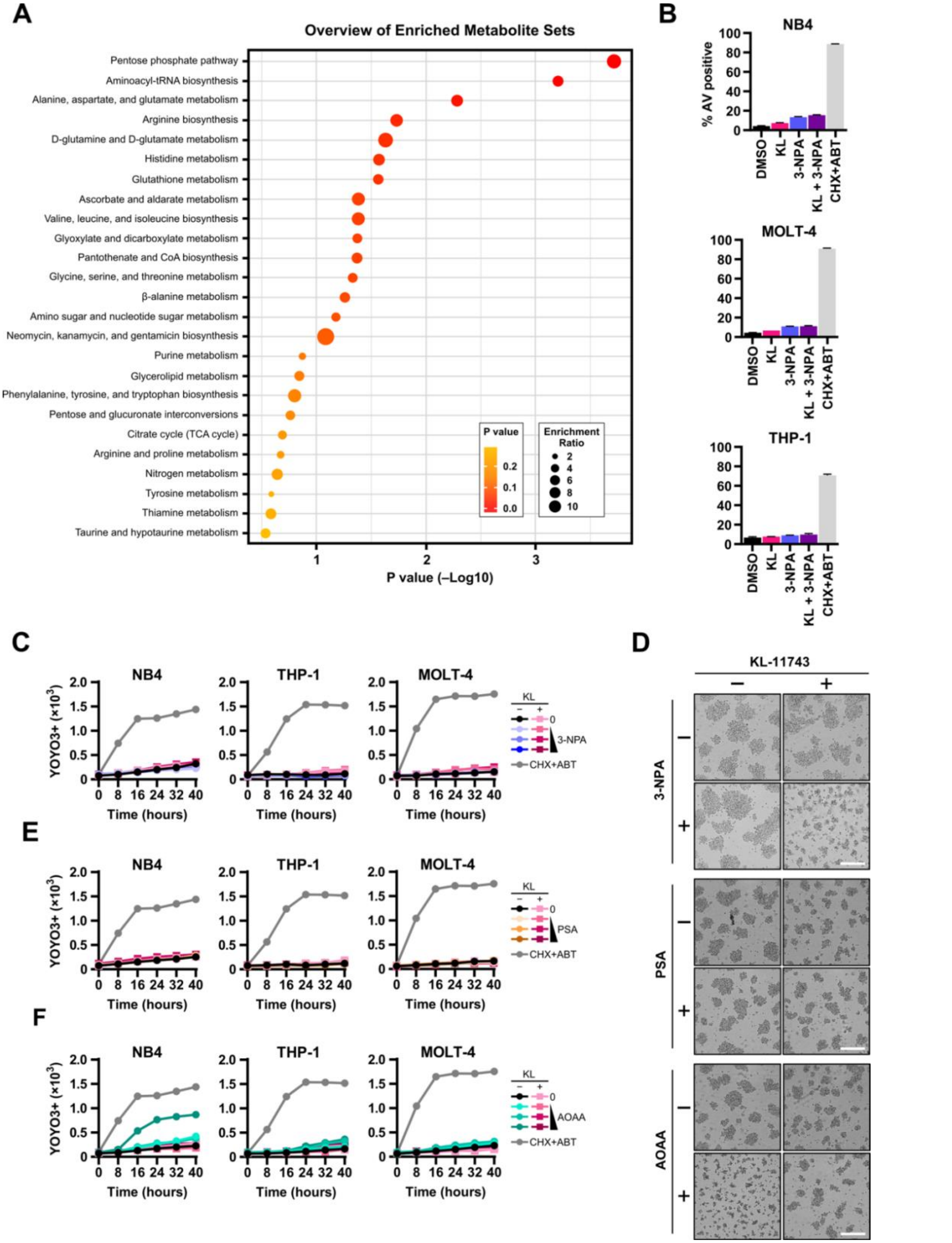

Supplemental Figure 3. KL-11743 does not induce a dependency on the malate-aspartate shuttle.

(A) Top 25 enriched metabolite sets in NB4 and MOLT-4 treated with KL-11743 (500 nM) for 24 h, compared to DMSO controls. P values were computed using an unpaired T test.

(B) NB4, THP-1, and MOLT-4 were treated with DMSO, KL-11743 (KL, 500 nM) ± 3-NPA (1 mM) for 24 h. Apoptosis was measured by AV labeling and flow cytometry. CHX (50 µg/mL) + ABT-737 (1 µM) is a positive control for apoptosis. Data are presented as mean values of at least 3 replicates ± SEM.

(C) NB4, THP-1, and MOLT-4 cells were treated with 3-NPA (0.5, 0.75, 1 mM) ± KL-11743 (500 nM), imaged every 8 h, and analyzed for YOYO3+ cells; the mean YOYO3+ events per image of 2 replicates is presented. CHX (50 µg/mL) + ABT-737 (1 µM) is a positive control for apoptosis.

(D) Representative images of NB4 treated with KL-11743 (500 nM) and/or 3-NPA (1 mM), phenylsuccinic acid (PSA, 1 mM), or aminooxyacetic acid (AOAA, 1mM) for 24 h. Scale bar: 300 µm.

(E) Same as *C*, but cells were treated with phenylsuccinic acid (PSA, 0.5, 0.75, 1 mM) ± KL-11743 (500 nM).

(F) Same as *C*, but cells were treated with aminooxyacetic acid (AOAA, 0.5, 0.75, 1 mM) ± KL-11743 (500 nM).

Supplemental Figure 4

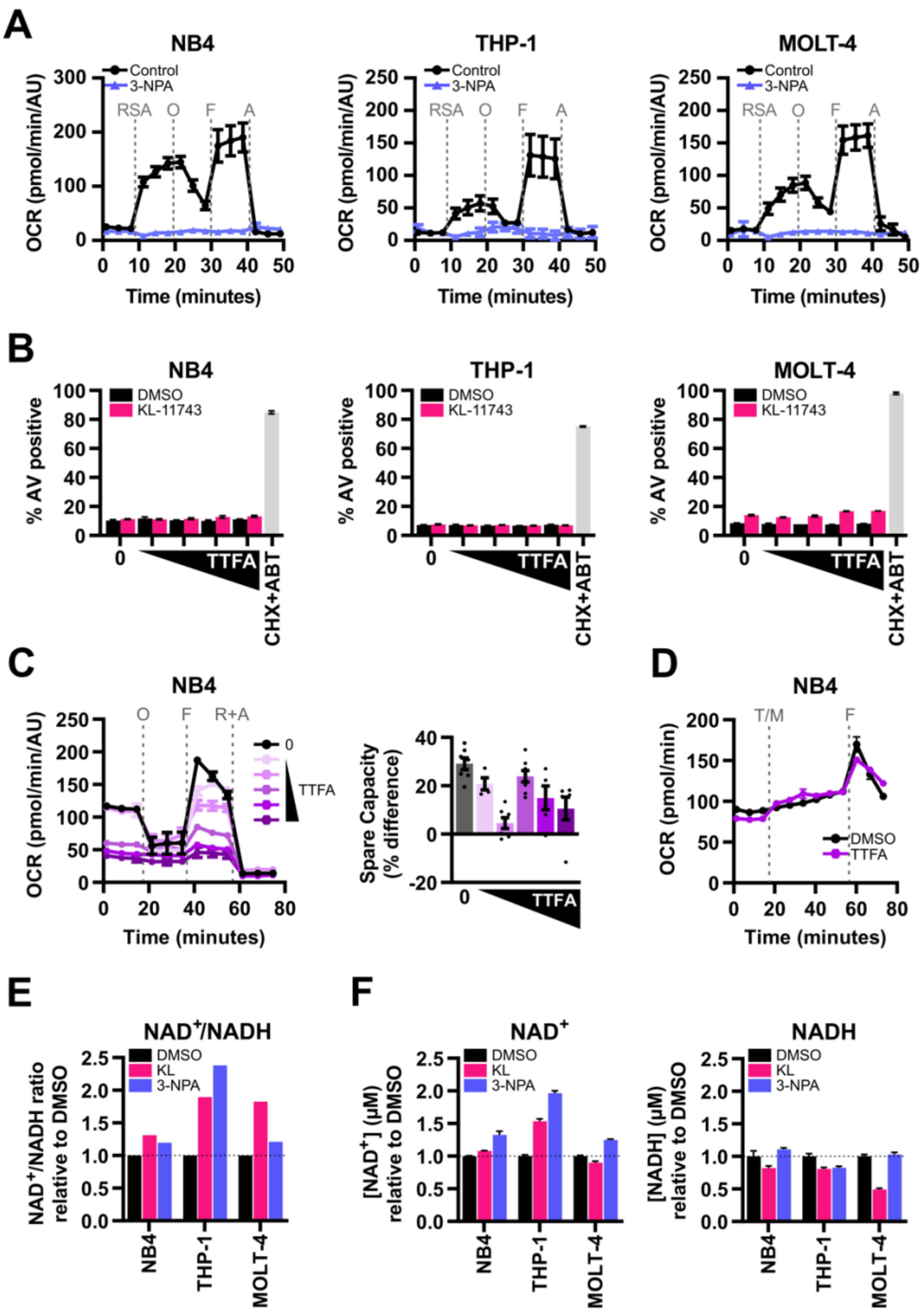

Supplemental Figure 4. Disruption of TCA cycle and inhibition of glycolysis both alter NAD^+/NADH redox.

(A) CII analysis of NB4, THP-1, and MOLT-4 treated with 3-NPA (1 mM) for 24 h. OCR were measured by an Agilent XFe96 Analyzer during sequential administration of a combination of RSA: rotenone (1 μM) + succinic acid (10 mM) + ADP (4 mM), O: oligomycin (1 μM), F: FCCP (1 μM), and A: antimycin A (0.5 μM).

(B) NB4, THP-1, and MOLT-4 were treated with DMSO, KL-11743 (500 nM), or TTFA (5, 10, 25, 50 μM) ± KL-11743 (500 nM) for 24 h. Apoptosis was measured by AV labeling and flow cytometry. CHX (50 μg/mL) + ABT-737 (1 μM) is a positive control for apoptosis. Data are the mean of 3 replicates ± SEM.

(C) Left: NB4 were treated with TTFA (10, 20, 30, 40, 50 μM) for 24 h and OCR was measured following an Agilent XF Cell Mito Stress Test. O: oligomycin (1 μM), F: FCCP (1 μM), R+A: rotenone (0.5 μM) + antimycin A (0.5 μM). Right: Quantification of spare respiratory capacity calculated as the percent difference between basal and maximal respiration reads.

(D) Changes in OCR of NB4 were measured using an Agilent XFe96 Analyzer after injection of TTFA (T, 50 μM) or XF RPMI medium (M) and FCCP (F, 1 μM).

(E–F) NB4, THP-1, and MOLT-4 were treated with DMSO, KL-11743 (500 nM), or 3-NPA (250 μM) for 24 h before NAD^+ and NADH concentrations were assessed. NAD^+/NADH ratios in *E* were determined using average NAD^+ and NADH concentrations from *F*.

Data are displayed as mean values of 3–6 technical replicates ± SEM.

Supplemental Figure 5

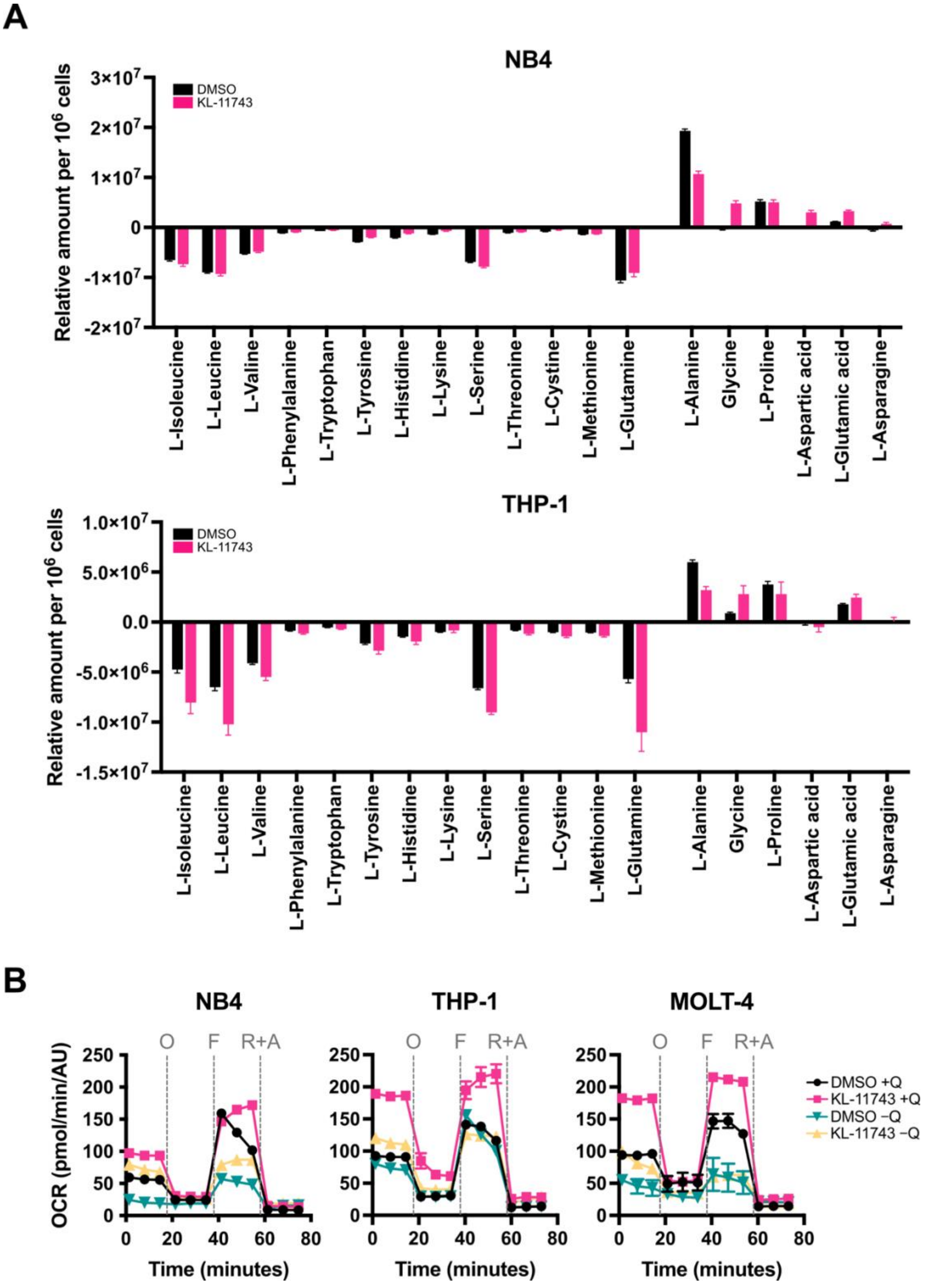

Supplemental Figure 5. KL-11743 shifts amino acid metabolism.

(A) NB4 and THP-1 were treated with DMSO or KL-11743 (500 nM) for 24 h, followed by GC-MS-EI media analysis of all amino acids. Average rates are graphed ± SEM, with negative values indicating consumption and positive values indicating secretion.

(B) NB4, THP-1, and MOLT-4 were treated with DMSO or KL-11743 (500 nM) for 24 h prior to changing them into glutamine-free ((-)Q) media for 2 h. OCR was measured using an Agilent XF Cell Mito Stress Test. O: oligomycin (1 μM), F: FCCP (1 μM), R+A: rotenone (0.5 μM) + antimycin A (0.5 μM).

Supplemental Figure 6

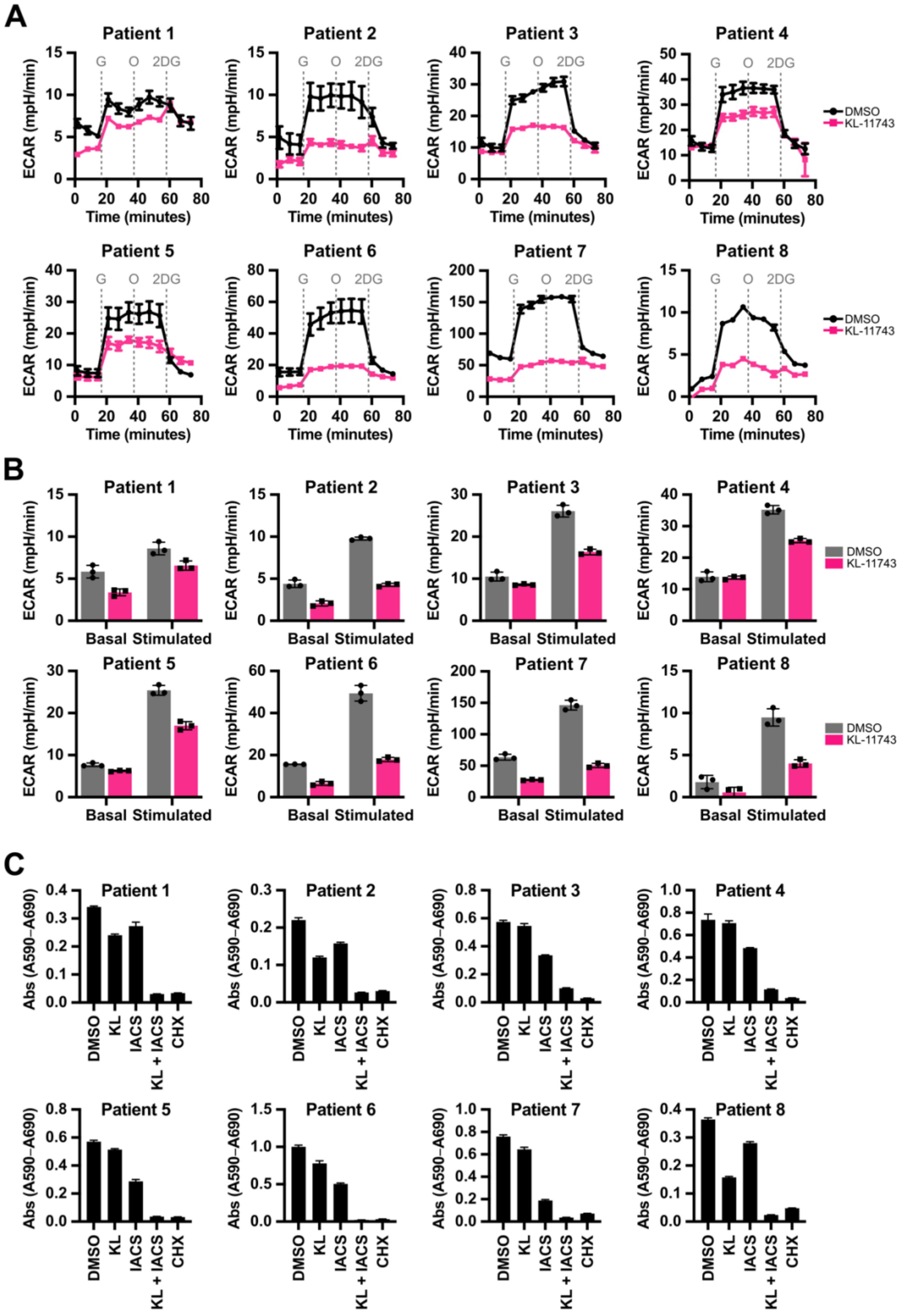

Supplemental Figure 6. AML patient mutations and expanded clinical data.

(A) Primary cells from AML patients 1–8 were treated with DMSO (0.1%) or KL-11743 (500 nM) for 24 h, and ECAR was measured following an Agilent XF Glycolysis Stress Test. G: glucose (10 mM), O: oligomycin (1 μM), 2DG: 2-deoxy-D-glucose (50 mM). All data are presented as mean values of at least 3 technical replicates ± SEM.

(B) Data from *A* presented as basal and glucose-stimulated ECAR. Data are presented as the mean of 3 technical replicates, averaged over the time points before or after glucose injection, ± SEM.

(C) Primary cells from AML patients 1–8 were treated with DMSO (0.1%), KL-11743 (500 nM), IACS (10 nM), CHX (50 μg/mL), or indicated combination for 24 h before viability was assessed by measuring the absorbance at 590 nm minus the reference absorbance at 690 nm. DMSO and CHX are the negative and positive cell death controls, respectively. All data are displayed as mean values of at least 6 technical replicates ± SEM.

Supplemental Figure 7

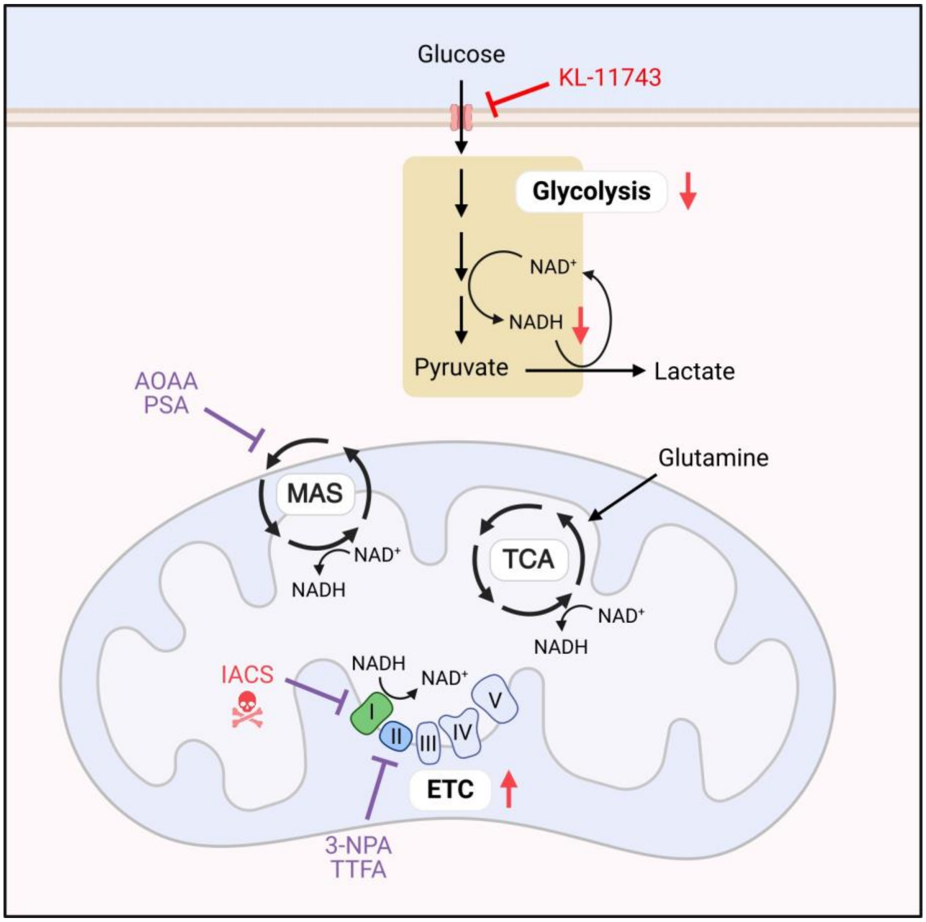

Supplemental Figure 7. Summary of KL-11743-induced metabolic changes.

KL-11743-mediated Class I glucose transporter (GLUT1–4) inhibition restricts glucose uptake, and results in: decreased glycolytic rate, lowering of cellular NADH pools, increased electron transport chain (ETC) activity, and higher mitochondrial oxygen consumption. Increased mitochondrial function relies on the presence of glutamine, and KL-11743 is synthetically lethal with IACS, an inhibitor of Complex I. Multiple inhibitors were used to characterize the cellular responses to KL-17743, including: inhibitors of the malate-aspartate shuttle (MAS): AOAA and PSA, and inhibitors of Complex II: 3-NPA and TTFA.
