# Supplementary figures and images for: Metabolic adaptations to acute glucose uptake inhibition converge upon mitochondrial respiration for leukemia cell survival
Source: Cell Commun Signal. 2025 Jan 25;23:47. doi: 10.1186/s12964-025-02044-y (PMC11762851; doi:10.1186/s12964-025-02044-y)

## Supplementary File 2

### Uncropped Gels for Figure 5A

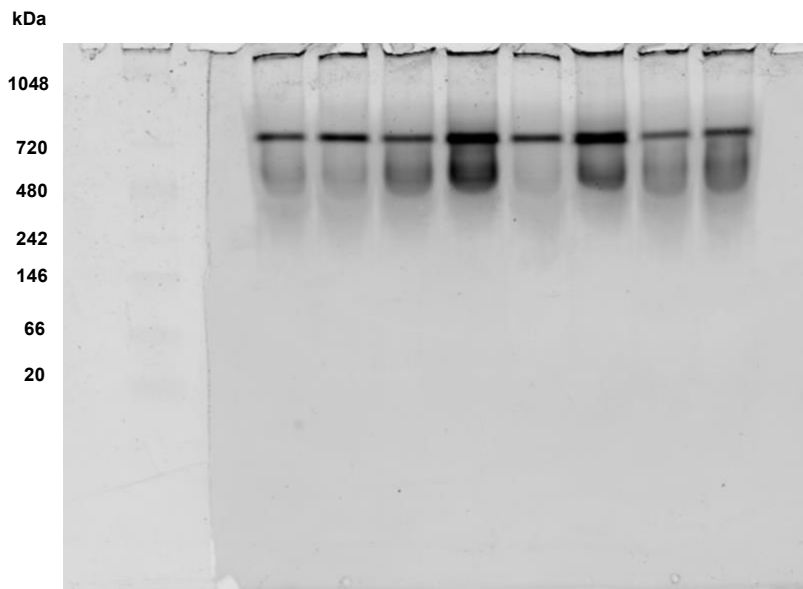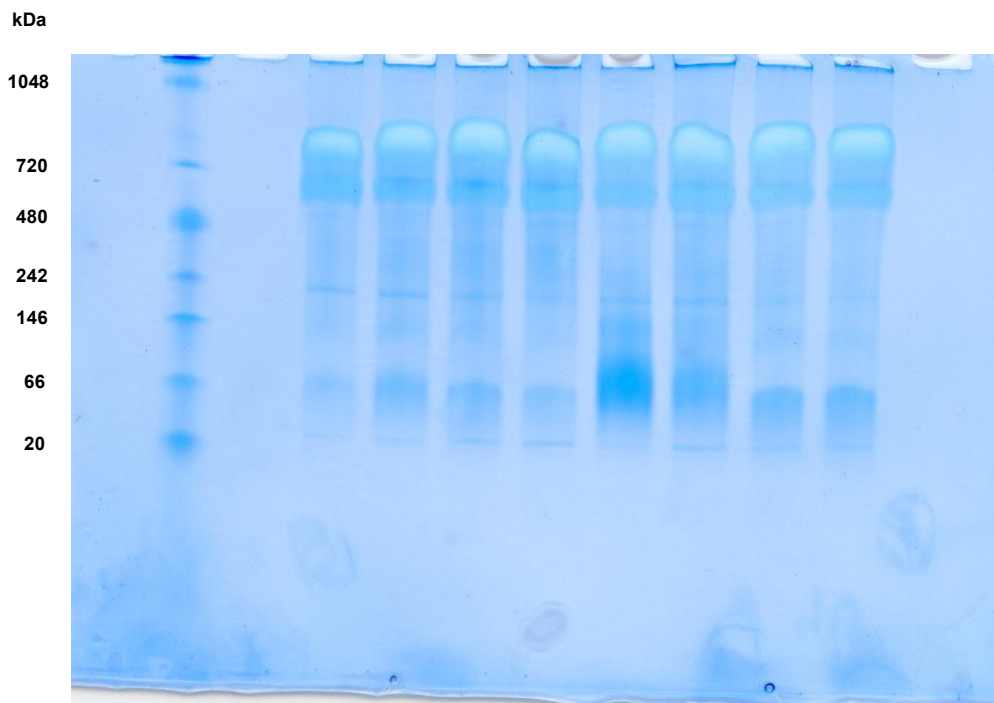

Supplement: Supplementary file 2 — Supplementary Material 2. [file 12964_2025_2044_MOESM2_ESM.pdf]
